# Builder-Blocker Mutual-Visibility Game

**DOI:** 10.1007/s40840-026-02083-9

**Published:** 2026-04-01

**Authors:** Vesna Iršič Chenoweth, Sandi Klavžar, Gregor Rus, Elif Tan, Jing Tian

**Affiliations:** 1 Faculty of Mathematics and Physics, University of Ljubljana, Ljubljana, Slovenia; 2 Institute of Mathematics, Physics and Mechanics, Ljubljana, Slovenia; 3 Faculty of Natural Sciences and Mathematics, University of Maribor, Maribor, Slovenia; 4 Department of Mathematics, Ankara University, Ankara, Türkiye; 5 School of Science, Zhejiang University of Science and Technolog, Hangzhou, Zhejiang 310023 PR China

**Keywords:** Mutual-visibility set, Games on graphs, Complete multipartite graph, Hamming graph, 05C12, 05C57, 05C69

## Abstract

This article discusses mutual-visibility in graphs through a game-based version of the problem. Two players, Builder and Blocker, alternately select an unmarked vertex on a graph keeping the property that the set of marked vertices forms a mutual-visibility set. The game ends when no such selection is possible. The goal of Builder is to create a largest possible mutual-visibility set, Blocker’s goal is the opposite. The central problem here is to determine the number of vertices selected during the game assuming that both players played optimally. Bounds on this number are proved and several general properties of the game derived. Special attention is paid to complete multipartite graphs and Hamming graphs.

## Introduction

Let $G = (V(G),E(G))$ be a connected graph. A set $X\subseteq V(G)$ is a *mutual-visibility set* if for every two vertices $x,y\in X$ there exists a shortest *x*, *y*-path such that none of the internal vertices of the path belongs to *X*. The *mutual-visibility number* $\mu (G)$ of *G* is the cardinality of a largest mutual-visibility set in *G*. Motivated by work in computer science, especially by the significance that mutual-visibility properties play in mobile entity models [1, 10, 22], these concepts were introduced into graph theory in 2022 [11], and immediately received a great deal of attention. As a selection of these studies, we should mention [6, 8, 9, 15, 18–20, 23, 24] .

The *Builder-Blocker mutual-visibility game* we are introducing in this article is defined as follows. There are two players, Builder and Blocker, who alternately select an unmarked vertex on a graph. There are two versions of the game, depending on who goes first. If Builder makes the first move, the game is shortly called the *B*-*game*, otherwise it is called the $B'$-*game*. At each step of each of the two games, the set of marked vertices must form a mutual-visibility set. The game ends when no more vertices can be selected. The goal of Builder is to create a largest possible mutual-visibility set, while the goal of Blocker is to keep the set as small as possible by blocking Builder. The number of vertices selected in the *B*-game, assuming that both players played optimally, is called the *Builder-game mutual-visibility number* of *G* and is denoted by $\mu _\textrm{g}(G)$. For the $B'$-game, this number is called the *Blocker-game mutual-visibility number* of *G* and is denoted by $\mu _\textrm{g}'(G)$.

An analogous game for the case of general position sets was investigated in [17]. Let’s emphasize that we are interested in the length of these games, which is closely related to the mutual-visibility number (resp. general position number). On the other hand, general position games in which we are only interested in the winner were discussed in [7, 16].

In the next section, several general properties of the Builder-Blocker mutual-visibility game are derived. In particular, the differences $\mu _\textrm{g}(G) - \mu _\textrm{g}'(G)$ and $\mu _\textrm{g}'(G) - \mu _\textrm{g}(G)$ can be arbitrarily large, which makes this game very different from the domination and related games [3]. Graphs *G* with $\mu _\textrm{g}(G) = 2$ and with $\mu _\textrm{g}'(G) = 2$ are also respectively described. In Section 3, complete multipartite graphs are studied. For $t\ge 3$, $r_1 \ge \cdots \ge r_t \ge 1$ and $r_1\ge 2$, it is derived that $\mu _\textrm{g}(K_{r_1, \ldots , r_t}) \in \{n-2, n-1\}$ and $\mu _\textrm{g}'(K_{r_1, \ldots , r_t}) \in \{n-2, n-1\}$, where $n = \sum _{i=1}^t r_i$. In the main result of Section 4 it is proved that $\mu _\textrm{g}(K_n{{\,\mathrm{\,\square \,}\,}}K_n) > n^{4/3}$ and that $\mu _\textrm{g}'(K_n{{\,\mathrm{\,\square \,}\,}}K_n) > n^{4/3}$. We conclude the article with several open problems that we find interesting for further research.

We now recall some standard definitions and notation used in the sequel. The order of *G* will be denoted by *n*(*G*) and the minimum and the maximum degree of *G* respectively by $\delta (G)$ and $\Delta (G)$. The neighborhood of a vertex *x* of *G* is denoted by *N*(*x*). The *girth* of *G* is the length of a shortest cycle of *G*; if *G* contains no cycles, its girth is infinite.

## Some General Properties

In [4], Brešar and Yero investigated the *lower mutual-visibility number* $\mu ^-(G)$ of a graph *G*, defined as the cardinality of a smallest maximal mutual-visibility set of *G*. Since at the end of the *B*-game and of the $B'$-game the set of selected vertices forms a maximal mutual-visibility set, we have

1 $$\begin{aligned} \mu ^-(G)&\le \mu _\textrm{g}(G) \le \mu (G), \end{aligned}$$

2 $$\begin{aligned} \mu ^-(G)&\le \mu _\textrm{g}'(G) \le \mu (G). \end{aligned}$$

For instance, it is known from the seminal paper [11, Lemma 2.8] that $\mu (C_n) = 3$, $n\ge 3$. It is also straightforward to check that $\mu ^-(C_n)= 3$, hence by (1) and (2) we can conclude that $\mu _\textrm{g}(C_n) = \mu _\textrm{g}'(C_n) = 3$, $n\ge 3$. From the game theoretic point of view, the lower bounds in (1) and (2) are achieved by the solitaire mutual-visibility game played by Blocker, while the upper bounds are achieved by the solitaire game played by Builder.

The proof of the following lemma is similar to that of  [17, Lemma 1]. For this reason we only provide a brief sketch.

### Lemma 2.1

Let *G* be a graph and consider the moment in a *B*-game or $B'$-game when *X* is the set of vertices played so far. If $X\cup X'$ is a mutual-visibility set, where $X'$ is the set of vertices which are playable as the next move, then the game will finish precisely after all the vertices from $X'$ have been played.

### Proof

By our assumption, a vertex from $V(G)\setminus (X\cup X')$ cannot be played at the current state of the game, hence it cannot be played in any of the later moves. On the other hand, since $X\cup X'$ is a mutual-visibility set, playing the vertices from $X'$ one by one makes a legal sequence of moves. $\square$

As an application of Lemma 2.1 we give:

### Theorem 2.2

The differences $\mu _\textrm{g}(G) - \mu _\textrm{g}'(G)$ and $\mu _\textrm{g}'(G) - \mu _\textrm{g}(G)$ can be arbitrarily large.

### Proof

**Fig. 1:**
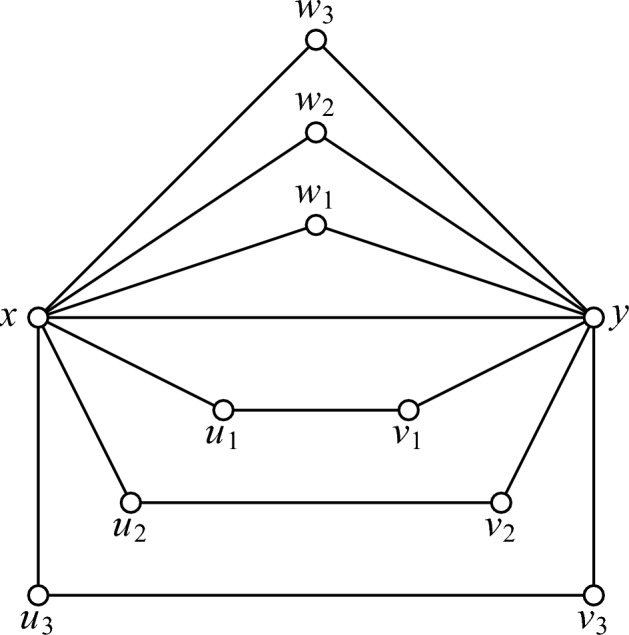
The graph $$G_3$$

We first demonstrate that the difference $\mu _\textrm{g}(G) - \mu _\textrm{g}'(G)$ can be arbitrarily large. For this sake consider the graphs $G_n$ obtained from *n* disjoint triangles and *n* disjoint $C_4$, by selecting an edge in each of these 2*n* graphs and identifying them into a single edge. See Fig. 1 where $G_3$ is shown. From the figure, the vertex labelling of $G_n$ should be clear.

Consider first the *B*-game. Let $w_1$ be the first move of Builder and assume that Blocker replies by selecting *x*. In this case, let the second move of Builder be $w_2$. After that, *y* is no longer playable for otherwise $w_1$ and $w_2$ are not visible. Because of that, all the vertices $w_2, \dots , w_n$ will be played by the end of the game. It follows that at least $n+1$ vertices are played in this case. Assume second that after the move $w_1$ of Builder, Blocker replies by selecting one of the vertices $u_i$ or $v_i$, we may assume it is the vertex $u_1$. Then Builder replies by *y*, and using a parallel argument as above, we see that at least $n+1$ vertices will be played. The last case to consider is if, without loss of generality, the first move of Blocker is $w_2$. Builder then selects *x* and we again have a game with at least $n+1$ vertices played. Hence $\mu _\textrm{g}(G_n)\ge n+1$.

Consider next the $B'$-game on $G_n$. In this game, Blocker can assure that after the first three moves of the game, both *x* and *y* are played. We can then check that in every such situation, the game is over, that is, $\mu _\textrm{g}'(G_n) = 3$. We have thus demonstrated that $\mu _\textrm{g}(G)$ can be arbitrarily larger than $\mu _\textrm{g}'(G)$.

For the reverse difference, consider the graphs $T_n$, $n\ge 2$, which are obtained from $K_2$ by attaching *n* leaves to each of the vertices of $K_2$. In the *B*-game, after Builder selects a vertex *u* of $T_n$, Blocker selects a neighbor of *u* to finish the game. Hence $\mu _\textrm{g}(T_n) = 2$. Consider next the $B'$-game. Then, no matter which vertex Blocker selects first, Builder can select a vertex such that at that point the two vertices selected are a vertex *x* of degree $n+1$ and a leaf *y* at distance two from *x*. Now, if $x'$ is the other vertex of degree $n+1$, then the set $S'$ of playable vertices at this moment are the $n-1$ leaves adjacent to $x'$ different from *y*. Since $\{x,y\}\cup S'$ is a mutual-visibility set, Lemma 2.1 implies that $\mu _\textrm{g}'(T_n) \ge n+1$. (One can also verify that $\mu _\textrm{g}'(T_n) \le n+1$.) We can conclude that $\mu _\textrm{g}'(G) - \mu _\textrm{g}(G)$ can be arbitrarily large. $\square$

**Fig. 2:**
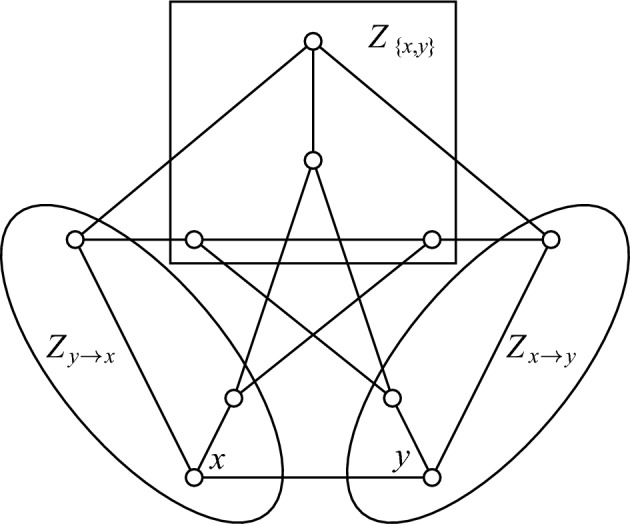
Petersen graph *P*, its edge *xy*, and sets $Z_{x\rightarrow y}$, $Z_{y\rightarrow x}$, $Z_{\{x,y\}}$

If *xy* is an edge of a connected graph *G*, then let $Z_{x\rightarrow y}$ denote the set of vertices *w*, such that every shortest *x*, *w*-path contains *y*, that is, every shortest *x*, *w*-path passes the edge *xy*. The set $Z_{y\rightarrow x}$ is defined analogously. Note that $y\in Z_{x\rightarrow y}$, $x\in Z_{y\rightarrow x}$, and $Z_{x\rightarrow y}\cap Z_{y\rightarrow x} = \emptyset$. Setting $Z_{\{x,y\}} = V(G) \setminus (Z_{x\rightarrow y}\cup Z_{y\rightarrow x})$ we thus infer that *V*(*G*) is the disjoint union of $Z_{\{x,y\}}$, $Z_{x\rightarrow y}$ and $Z_{y\rightarrow x}$. An example illustrating these sets can be seen in Fig. 2.

The following result is a variant of [17, Theorem 1] for the Builder-Blocker mutual-visibility.

### Theorem 2.3

If *G* is a connected graph with $n(G)\ge 2$, then

$$\begin{aligned} \mu _\textrm{g}(G) \le 2 + \max _{x\in V(G)} \min _{y\in N(x)} \left| Z_{\{x,y\}}\right| \,. \end{aligned}$$

### Proof

Let *x* be the first vertex selected by Builder in the *B*-game. To prove the upper bound, let’s consider a strategy of Blocker in which they reply by selecting a neighbor *y* of *x*. Then, by the definition of the sets $Z_{x\rightarrow y}$ and $Z_{y\rightarrow x}$, no vertex from their union can be played during the rest of the game. It follows that at most $2 + |Z_{\{x,y\}}|$ vertices will be selected under the assumption that *x* and *y* were played first. Now, as Builder starts the game and wishes to maximize the number of vertices selected, the bound follows. $\square$

### Corollary 2.4

If *G* is a connected graph of girth at least 5, then

$$\begin{aligned} \mu _\textrm{g}(G) \le n(G) - 2\delta (G) + 2\,. \end{aligned}$$

### Proof

Let $xy\in E(G)$. Since the girth of *G* is at least 5 we have $N(x)\cap N(y) = \emptyset$. From the same reason, $N(y) \subseteq Z_{x\rightarrow y}$ and $N(x) \subseteq Z_{y\rightarrow x}$. If follows that

$$\begin{aligned} |Z_{\{x,y\}}|&= n(G) - |Z_{x\rightarrow y}| - |Z_{y\rightarrow x}| \\&\le n(G) - |N(y)| - |N(x)| \\&\le n(G) - 2\delta (G). \end{aligned}$$

Since *xy* is an arbitrary edge of *G*, Theorem 2.3 implies the assertion. $\square$

For the Petersen graph *P*, Corollary 2.4 gives $\mu _\textrm{g}(P)\le 6$. On the other hand, $\{x,y\}\cup Z_{\{x,y\}}$ is a mutual-visibility set, hence Lemma 2.1 yields $\mu _\textrm{g}(P)\ge 6$. We can conclude that $\mu _\textrm{g}(P) = 6$ which in turn implies that the bound of Corollary 2.4 is sharp.

*Geodetic graphs* are the graphs with the property that every two vertices are joined by a unique shortest path. For instance, trees, block graphs, and *k*-trees are examples of geodetic graphs, as well as it is also the Petersen graph. This concept goes back all the way to Ore [21], an early survey is given in [2]. We also refer to [12, 14] for selected recent developments. From our perspective, the following property of geodetic graphs is relevant, where $\textrm{gp}_\textrm{g}(G)$ and $\textrm{gp}_\textrm{g}'(G)$ respectively denote the Builder-game general position number and the Blocker-game general position number [17].

### Proposition 2.5

If *G* is a geodetic graph, then $\mu _\textrm{g}(G) = \textrm{gp}_\textrm{g}(G)$ and $\mu _\textrm{g}'(G) = \textrm{gp}_\textrm{g}'(G)$.

### Proof

Since *G* is geodetic, a set $X\subseteq V(G)$ is a mutual-visibility set if and only if *X* is a general position set. The admissible sets therefore coincide in the Builder-Blocker mutual-visibility game and the Builder-Blocker general position game played on *G*, hence the conclusions. $\square$

By Proposition 2.5, all the results from [17] derived for $\textrm{gp}_\textrm{g}(T)$ and $\textrm{gp}_\textrm{g}'(T)$, where *T* is a tree, immediately apply to $\mu _\textrm{g}(T)$ and $\mu _\textrm{g}'(T)$, respectively. For instance, if $\ell (T)$ denotes the number of leaves of a tree *T*, then $\mu _\textrm{g}'(T)\le \ell (T) - \Delta (T) + 2$. Moreover, it follows from [17, Corollary 2] that $\mu _\textrm{g}(T) = 2$ for every tree *T* on at least two vertices. This result generalizes as follows, where by a *starlike tree* we mean a tree which contains at most one vertex of degree at least 3.

### Proposition 2.6

If *G* is a connected graph of order at least two, then the following assertions hold.

(i) $\mu _\textrm{g}(G) = 2$ if and only if *G* is a tree.
(ii) $\mu _\textrm{g}'(G) = 2$ if and only if *G* is a starlike tree.

### Proof

(i) As already mentioned, [17, Corollary 2] implies $\mu _\textrm{g}(T) = 2$ for every tree *T*. Assume now that *G* is not a tree. If *C* is a shortest cycle of *G*, then let Builder select a vertex of *C* as their first move. Since *C* is isometric, no matter whether Blocker plays a vertex of *C* or not as their first move, Builder is able to play at least one more move. Hence $\mu _\textrm{g}(G) \ge 3$ if *G* is not a tree.

(ii) If *T* is a path, then clearly $\mu _\textrm{g}'(T) = 2$. If *T* is a starlike tree which is not a path, then *T* contains a vertex *w* of degree at least 3. Then Blocker starts the $B'$-game by playing *w* and no matter which vertex is played by Builder, the game is over afterwards. Assume next that *T* is a tree with at least two vertices of degree at least 3, say *w* and $w'$. Let *x* be an optimal first move of Blocker. Let $T_w$ be the maximal subtree of *T* that contains *w* and does not contain the edges on the $w,w'$-path. Define the subtree $T_{w'}$ analogously. Then we may without loss of generality assume that $x\notin V(T_w)$. Now, if Builder plays a leaf of $T_w$, then at least one more vertex will be played, which implies $\mu _\textrm{g}'(T)\ge 3$. Consider finally a graph *G* containing a cycle. Then considering a shortest cycle *C* of *G* again, we can infer that Builder will be able to force Blocker to play at least two moves, hence also now $\mu _\textrm{g}'(G)\ge 3$. $\square$

## Complete Multipartite Graphs

In this section, we investigate the Builder-Blocker mutual-visibility game on complete multipartite graphs. Since the mutual-visibility number of the complete bipartite graphs $K_{m,n}$ for $n\ge m\ge 3$ has already been determined in [11, Theorem 4.9], we focus here on the complete multipartite graphs $K_{r_1, \ldots , r_t}$, $t\ge 3$.

### Lemma 3.1

If $r_1 \ge \cdots \ge r_t \ge 1$ and $n = \sum _{i=1}^t r_i$, then

$$\mu (K_{r_1, \ldots , r_t}) = {\left\{ \begin{array}{ll} n; & \text { if } r_1 = 1, \\ n-1; & \text { if } r_1 \ge 2 \textrm{and}\ r_t \le 2, \\ n-2; & \text {if } r_t \ge 3. \\ \end{array}\right. }$$

### Proof

Let $V(K_{r_1, \ldots , r_t}) = \bigcup _{i = 1}^{t} X_i$, where $X_i = \{x_{i1},\ldots ,x_{ir_i}\}$ for $i\in [t]$. If $r_1 = \cdots = r_t = 1$, then $K_{r_1, \ldots , r_t}$ is a complete graph of order *t* for which we have $\mu (K_t) = t$. In the rest of the proof hence assume that $r_1\ge 2$.

Assume first that $r_t\le 2$, and let $X = \bigcup _{i = 1}^{t-1} X_i\cup (X_t\setminus \{x_{t1}\})$. Consider any two vertices *u* and *v* from $K_{r_1, \ldots , r_t}$. If $u\in \bigcup _{i = 1}^{t-1} X_i$ and $v\in (X_t\setminus \{x_{t1}\})$, then *u* is adjacent to *v* and we see that *u* and *v* are *X*-visible. If $u,v\in \bigcup _{i = 1}^{t-1} X_i$, the vertices *u*, *v*, and $x_{t1}$ induce a shortest *u*, *v*-path of length 2, hence *u* and *v* are *X*-visible. Then *X* is a mutual-visibility set of $K_{r_1, \ldots , r_t}$, therefore $\mu (K_{r_1, \ldots , r_t}) \ge n-1$. On the other hand, since $r_1\ge 2$, we see that $K_{r_1, \ldots , r_t}$ is not complete, hence $\mu (K_{r_1, \ldots , r_t}) \le n-1$.

Assume second that $r_t\ge 3$, and let $X' = \bigcup _{i = 1}^{t} X_i\setminus \{ x_{11},x_{t1}\}$. Using similar arguments as to the above, $X'$ is a mutual-visibility set of $K_{r_1, \ldots , r_t}$, hence $\mu (K_{r_1, \ldots , r_t})\ge n-2$. On the other hand, since $r_1 \ge \cdots \ge r_t \ge 3$ we infer that no set of cardinality $n-1$ can be a mutual-visibility set. It follows that $\mu (K_{r_1, \ldots , r_t})\le n-2$ and we are done. $\square$

We now analyze the Builder-Blocker mutual-visibility game on the complete multipartite graph $K_{r_1, \ldots , r_t}$. If $r_1 = \cdots = r_t = 1$, then we deal with $K_t$ for which we clearly have $\mu _\textrm{g}(K_t) = \mu _\textrm{g}'(K_t) = t$. For the case when $t=2$ we have the following.

### Theorem 3.2

If $n\ge m$, then the following assertions hold.

(i) If $$m =1$$, then $$\mu _\textrm{g}(K_{m,n}) = \mu _\textrm{g}'(K_{m,n}) = 2$$.
(ii) If $m = 2$, then $\mu _\textrm{g}(K_{m,n}) =n +1$ and $\mu _\textrm{g}'(K_{m,n}) = 3$.
(iii) If $m\ge 3$, then $\mu _\textrm{g}(K_{m,n}) = \mu _\textrm{g}'(K_{m,n}) = m +n -2$.

### Proof

Let *M* and *N* be the (bipartition) parts of $K_{m,n}$, where $|M| = m$ and $|N| = n$.

(i) In this case, $K_{m,n}$ is a tree, thus by the comments made after Proposition 2.5, $\mu _\textrm{g}(K_{m,n}) = 2$ and $\mu _\textrm{g}'(K_{m,n}) \le n-n+2 = 2$. As $\mu ^{-}(K_{m,n}) = 2$ (take the central vertex and one leaf), the conclusion follows by (2).

(ii) In the *B*-game, Builder’s strategy is to first select a vertex from *N*, and also play on *N* in the second move, unless Blocker plays on *N* in the first move. As two vertices of *N* are then selected, only one vertex from *M* can be selected during the game. Thus the game ends when all the vertices from *N* and one vertex from *M* are played. Thus $\mu _\textrm{g}(K_{m,n}) \ge n+1$. On the other hand, Lemma 3.1 gives $\mu (K_{m,n}) = n+m-1=n+1$, thus (1) concludes the argument in this case.

Consider next the $B'$-game. As $\mu ^{-}(K_{m,n}) = 3$ (take both vertices from *M* and then only one vertex from *N* can be added), $\mu _\textrm{g}'(K_{m,n}) \ge 3$ by (2). Blocker’s strategy to achieve this is to start the game by playing a vertex from *M*, and also playing on *M* in the second move (unless Builder does in the first move). Hence $\mu _\textrm{g}'(K_{m,n}) \le 3$.

(iii) By Lemma 3.1 and (1), $\mu _\textrm{g}(K_{m,n}) \le m+n-2$. To achieve this, Builder’s strategy is to force at least two vertices from *M* and at least two from *N* to be played before all vertices are played from either part of the bipartition. To achieve this in the *B*-game, Builder starts by playing a vertex from *N*, and afterwards plays in the opposite part of where Blocker plays.

In the $B'$-game, Builder plays in the opposite part as Blocker, thus after the second move of Builder, two vertices have been played in *M* and two in *N*. Now, all but one vertex from each part of the bipartition can be selected until the end of the game, therefore the game lasts at least $m+n-2$ moves. $\square$

We now turn our attention to the general case of complete multipartite graphs $K_{r_1, \ldots , r_t}$ for $t\ge 3$ and $r_1\ge 2$.

### Theorem 3.3

If $t \ge 3$, $r_1 \ge \cdots \ge r_t \ge 1$, $r_1\ge 2$, and $n = \sum _{i=1}^t r_i$, then

$$\begin{aligned} n-2 \le \mu _\textrm{g}(K_{r_1, \ldots , r_t}) \le n-1. \end{aligned}$$

### Proof

Since $t\ge 3$ and $r_1\ge 2$, the upper bound follows by Lemma 3.1 and (1). To prove the lower bound we need a strategy of Builder that ensures that at least $n-2$ moves are played during the game. First observe that lower mutual-visibility sets of $K_{r_1, \ldots , r_t}$ are of the following form: all vertices except from one part of the partition which contributes only one vertex to the set (or zero if this part is of size 1), or all vertices except two vertices that belong to different parts of the partition. As the lower mutual-visibility number is a lower bound for $\mu _\textrm{g}(K_{r_1, \ldots , r_t})$, the lower bound does not trivially follow only if $r_1 \ge 4$ and Blocker can force the game mutual-visibility set to be all vertices except only one from a part of size at least 4. Thus let $1 \le k \le t$ be such that $r_k \ge 4$ and $r_{k+1} \le 3$. Builder’s strategy is to play two vertices in each part of size $r_1, \ldots , r_k$ (in this order). If Builder is able to play the second vertex in the part of size $r_k$, then the game will last for at least $n-2$ moves. Blocker is not able to prevent Builder from playing the second move in the part of size $r_k$ if and only if

$$\begin{aligned} (r_1-2) + \cdots + (r_{k-1}-2) + r_{k+1} + \cdots + r_t \le 2k-1, \end{aligned}$$

which simplifies to

$$\begin{aligned} 2(k-1) + (t-k) \le 2k-1, \end{aligned}$$

thus

$$\begin{aligned} k \le t \le k+1. \end{aligned}$$

If $t = k$, then the above bound gives

$$\begin{aligned} (r_1-2) + \cdots + (r_{k-1}-2)&\le 2k-1, \\ r_1 + \cdots + r_{k-1}&\le 4k-3, \end{aligned}$$

thus the only two possibilities are $r_1 = 5, r_2 = \cdots =r_t = 4$ and $r_1 = \cdots = r_t = 4$.

If $t=k+1$, then the above bound gives

$$\begin{aligned} (r_1-2) + \cdots + (r_{k-1}-2) + r_t&\le 2k-1, \\ r_1 + \cdots + r_{k-1} + r_t&\le 4k-3, \end{aligned}$$

thus the only possibility is $r_1 = \cdots = r_{t-1} = 4, r_t = 1$.

Note that $t\ge 3$. Once there exist two parts of size 4 in $K_{r_1, \ldots , r_t}$, Builder can adopt a strategy that at least two vertices are played in each in such part. The game will end after at least $n-2$ vertices have been played. We can conclude that $\mu _\textrm{g}(K_{r_1, \ldots , r_t})\ge n-2$. $\square$

By the proof of Theorem 3.3 and by Lemma 3.1, we obtain the following:

### Corollary 3.4

If $t \ge 3$, $r_1 \ge \cdots \ge r_t \ge 3$, and $n = \sum _{i=1}^t r_i$, then

$$\begin{aligned} \mu _\textrm{g}(K_{r_1, \ldots , r_t}) = n-2. \end{aligned}$$

For the $B'$-game played on $K_{r_1, \ldots , r_t}$, where $t\ge 3$ and $r_1\ge 2$, we have:

### Theorem 3.5

If $t \ge 3$, $r_1 \ge \cdots \ge r_t \ge 1$, $r_1\ge 2$ and $n = \sum _{i=1}^t r_i$, then

$$\begin{aligned} n-2 \le \mu _\textrm{g}'(K_{r_1, \ldots , r_t}) \le n-1. \end{aligned}$$

### Proof

As $t\ge 3$ and $r_1\ge 2$, Lemma 3.1 and (2) provide the upper bound. To prove the lower bound we need a strategy of Builder that ensures that at least $n-2$ moves are played during the game. The idea is similar as in the proof of Theorem 3.3, here we omit the details. $\square$

### Corollary 3.6

If $t \ge 3$, $r_1 \ge \cdots \ge r_t \ge 3$, and $n = \sum _{i=1}^t r_i$, then

$$\begin{aligned} \mu _\textrm{g}'(K_{r_1, \ldots , r_t}) = n-2. \end{aligned}$$

## Hamming Graphs

Let *G* and *H* be two graphs. The Cartesian product $G{{\,\mathrm{\,\square \,}\,}}H$ has the vertex set $V(G)\times V(H)$, vertices (*g*, *h*) and $(g',h')$ being adjacent if either $g=g'$ and $hh'\in E(H)$, or $h=h'$ and $gg'\in E(G)$.

In this section we consider the Builder-Blocker mutual-visibility game on the Cartesian product of two complete graphs, also known as Hamming graphs. For the mutual-visibility of the Cartesian product of two complete graphs, the following result is crucial.

### Lemma 4.1

[8, Lemma 3.5] Let $n,m\ge 2$ and let $X\subseteq V(K_n{{\,\mathrm{\,\square \,}\,}}K_m)$. Then *X* is a mutual-visibility set of $K_n{{\,\mathrm{\,\square \,}\,}}K_m$ if and only if $|X\cap V(C)|\le 3$ holds for each induced 4-cycle *C* of $K_n{{\,\mathrm{\,\square \,}\,}}K_m$.

By Lemma 4.1, inequalities (1) and (2), and [8, Corollary 3.7], we deduce that

$$\begin{aligned} n + m - 1&\le \mu _\textrm{g}(K_n{{\,\mathrm{\,\square \,}\,}}K_m) \le z(n,m; 2,2)\,, \\ n + m - 1&\le \mu _\textrm{g}'(K_n{{\,\mathrm{\,\square \,}\,}}K_m) \le z(n,m; 2,2)\,, \end{aligned}$$

for any $m,n\ge 2$, where *z*(*m*, *n*; 2, 2) denotes the maximum number of 1s in a $m\times n$ binary matrix which contains no $2\times 2$ submatrix consisting of four 1s. The exact value of *z*(*m*, *n*; 2, 2) is widely open, cf [25], but the following bounds are known.

### Theorem 4.2

[5, 13, Brown, 1966; Erdős-Rényi-Sós, 1966] When n is sufficiently large,

$$\begin{aligned} n^{3/2}-n^{4/3} \le z(n,n;2,2) \le \frac{1}{4} n(1+ \sqrt{4n-3})\,. \end{aligned}$$

The following result completes Theorem 4.2 for the case of the Builder-Blocker mutual-visibility games.

### Theorem 4.3

If $n\ge 2$, then $\mu _\textrm{g}(K_n{{\,\mathrm{\,\square \,}\,}}K_n) > n^{4/3}$ and $\mu _\textrm{g}'(K_n{{\,\mathrm{\,\square \,}\,}}K_n) > n^{4/3}$.

### Proof

An induced 4-cycle of $K_n{{\,\mathrm{\,\square \,}\,}}K_n$ projects on each of the two factors onto an edge, hence $K_n{{\,\mathrm{\,\square \,}\,}}K_n$ contains $$\left( {\begin{array}{c}n\\ 2\end{array}}\right) \left( {\begin{array}{c}n\\ 2\end{array}}\right) $$ induced 4-cycles. If $X\subseteq V(K_n{{\,\mathrm{\,\square \,}\,}}K_n)$, then we say that an induced 4-cycle *C* is *hit* by *X* if $|V(C)\cap X|\ge 3$. Since each triple of vertices of *X* can hit at most one induced 4-cycle, it follows that *X* hits at most $$\left( {\begin{array}{c}|X|\\ 3\end{array}}\right) $$ induced 4-cycles of $K_n{{\,\mathrm{\,\square \,}\,}}K_n$.

Assume now that *X* is the set of vertices selected so far by Builder and Blocker in the *B*-game or in the $B'$-game, and set $k = |X|$. If the number of induced 4-cycles hit by the vertices from *X* is smaller than the number of all induced 4-cycles, then by Lemma 4.1, in an arbitrary not yet hit induced 4-cycle, at least one vertex is playable, that is, the game is not over yet. That is, if

3 $$\begin{aligned} \left( {\begin{array}{c}k\\ 3\end{array}}\right) < \left( {\begin{array}{c}n\\ 2\end{array}}\right) \left( {\begin{array}{c}n\\ 2\end{array}}\right) \,, \end{aligned}$$

then $\mu _\textrm{g}(K_n{{\,\mathrm{\,\square \,}\,}}K_n) > k$ and $\mu _\textrm{g}'(K_n{{\,\mathrm{\,\square \,}\,}}K_n) > k$. Inequality (3) rewrites as

4 $$\begin{aligned} k(k-1)(k-2) - \frac{3}{2}n^2(n-1)^2 < 0\,. \end{aligned}$$

Setting $k = n^{4/3}$ we infer that (4) holds true for each $n\ge 2$. We can conclude that if $k = n^{4/3}$, then the games are not over yet. $\square$

## Concluding Remarks

In this section we collect some problems that seem interesting for further research and give some remarks on each of them.

From [8, Remark 4.5] we know that if *G* is a block graph, then $\mu (G) = s(G)$, where *s*(*G*) is the number of simplicial vertices of *G*. In addition, in [4, Theorem 14] it is proved that if *G* is a connected block graph with $n(G)\ge 2$, and *Q* is a maximal clique with minimum cardinality in *G*, then $\mu ^-(G) = |Q|$. This discussion leads to:

### Problem 5.1

Determine $\mu _\textrm{g}(G)$ and $\mu _\textrm{g}'(G)$ where *G* is a block graph.

In [11], $\mu (K_{m,n})$ is determined for all possible values of *m* and *n*. In Section 3 we extend this result to complete multipartite graphs. In the same section upper and lower bounds for $\mu _\textrm{g}$ and $\mu _\textrm{g}'$ of the latter graphs are given that differ by 1, and an exact result is provided for the case when we have at least three parts and all parts contain at least three vertices. It would be interesting to obtain exact values for the remaining complete multipartite graphs, that is:

### Problem 5.2

Characterize complete multipartite graphs $K_{r_1, \ldots , r_t}$ with $\mu _\textrm{g}(K_{r_1, \ldots , r_t}) = n-1$ and those with $\mu _\textrm{g}'(K_{r_1, \ldots , r_t}) = n-1$.

By [11, Theorem 4.6] we have $\mu (P_n{{\,\mathrm{\,\square \,}\,}}P_m) = 2\min \{n,m\}$ for $n,m\ge 4$, and by [4, Corollary 4] we have $\mu ^-(P_n{{\,\mathrm{\,\square \,}\,}}P_m) = 3$ for $n,m\ge 2$. Therefore, if $n \ge m\ge 4$, then

$$\begin{aligned} 3 \le \mu _\textrm{g}(P_n{{\,\mathrm{\,\square \,}\,}}P_m) \le 2m\,. \end{aligned}$$

We note that $\mu _\textrm{g}(P_3{{\,\mathrm{\,\square \,}\,}}P_3) = \mu _\textrm{g}'(P_3{{\,\mathrm{\,\square \,}\,}}P_3) = 4$; the strategy of Blocker is to first play the central vertex (unless Builder already played it). Moreover, $\mu _\textrm{g}(P_3{{\,\mathrm{\,\square \,}\,}}P_m) = \mu _\textrm{g}'(P_3{{\,\mathrm{\,\square \,}\,}}P_m) = 6$ as soon as *m* is large enough; the strategy of Builder is always to play in the same $P_3$-layer as Blocker and this is as far from Blocker as possible to make enough space. For the general case we pose:

### Problem 5.3

For any *n* and *m*, determine $\mu _\textrm{g}(P_n{{\,\mathrm{\,\square \,}\,}}P_m)$ and $\mu _\textrm{g}'(P_n{{\,\mathrm{\,\square \,}\,}}P_m)$.

In view of Problem 5.3 and Theorem 4.3 we also pose:

### Problem 5.4

For arbitrary connected graphs *G* and *H*, derive general upper and lower bounds on $\mu _\textrm{g}(G{{\,\mathrm{\,\square \,}\,}}H)$ and $\mu _\textrm{g}'(G{{\,\mathrm{\,\square \,}\,}}H)$.

## Data Availability

Data sharing is not applicable to this article as no new data were created or analyzed in this study.
